# Shoot transcriptome and co-expression network analysis of African rice cultivars identify drought-tolerance hub genes and growth stage-dependent trade-offs

**DOI:** 10.3389/fpls.2026.1802527

**Published:** 2026-04-15

**Authors:** Kossi Lorimpo Adjah, Mawuli Aziadekey, Aboubacar Toure, Maxwell Darko Asante, Isaac Tawiah, Shailesh Yadav, Geoffrey Onaga, Nana Kofi Abaka Amoah, Raafat El-Namaky, Komi Agboka, Rossana Henriques

**Affiliations:** 1 Africa Rice Center (AfricaRice), M’be Research Station, Bouaké, Côte d’Ivoire; 2 West African Science Service Center on Climate Change and Adapted Land Use/Climate Change and Agriculture Program, IPR/IFRA, Katibougou, Mali; 3 University of Sciences, Techniques and Technologies of Bamako (USTTB), Bamako, Mali; 4 High School of Agriculture, University of Lomé (UL), Lomé, Togo; 5 School of Biological, Earth and Environmental Sciences, University College Cork, Cork, Ireland; 6 International Crops Research Institute for the Semi-Arid Tropics (ICRISAT), Bamako, Mali; 7 Council for Scientific and Industrial Research-Crops Research Institute (CSIR-CRI), Fumesua- Kumasi, Ghana; 8 Africa Rice Center (AfricaRice), Sahel Regional Station, Saint-Louis, Senegal; 9 Sustainability Institute, Cork, Ireland

**Keywords:** differentially expressed genes, drought-stress, rice, RNA-Seq, RT-qPCR

## Abstract

Drought is a major abiotic stress limiting rice growth and productivity, posing a considerable threat to global food security. Understanding the molecular mechanisms that regulate plant responses to water deficit is critical for developing drought-tolerant rice varieties. While most breeding programmes evaluate drought responses at the adult or reproductive stages, early developmental responses remain understudied. Here, we compared the responses of young rice plants from previously characterized drought-tolerant (APO and CRI-Enapa) and drought-sensitive (ART132-35-1-1-B-B and CRI-Amankwatia) rice varieties. We applied a transcriptome-based weighted gene co-expression network analysis using WGCNA to identify key regulatory genes and pathways associated with drought response in rice. Comprehensive transcriptional profiling after 30 days of drought stress revealed that APO showed extensive transcriptional reprogramming with 96.63% and 97.32% uniquely differentially expressed genes (DEGs) compared to CRI-Enapa and ART132-35-1-1-B-B, respectively. Module-trait relationship analysis identified several modules significantly associated with shoot fresh weight and root fresh weight under drought stress and control condition, with the turquoise and blue modules showing the strongest correlations. Within these, 30 genes exhibited exceptionally high connectivity, suggesting potential central roles in the regulatory network. Notably, *S-acyltransferase* (*BGIOSGA023969*) and *NAD(P)-binding Rossmann-fold protein* (*BGIOSGA038191*) showed the highest correlation with the shoot and root fresh weight. Functional enrichment analyses of APO and hub genes revealed that most of the DEGs were associated with phytohormone signalling, transcription factors, carbohydrate metabolism and drought response genes, suggesting their key role in drought tolerance mechanisms. These transcriptional units may not only serve as potential targets for functional validation but also function as molecular markers for drought tolerance at the early-developmental stages, which is critical for successful crop establishment in stressful paddy environments.

## 1 Introduction

Rice is the staple food for more than 50% people on the planet Earth (Aslam et al., 2022). Despite its crucial role in food security, rice cultivation is heavily dependent on freshwater resources due to its limited adaptability to less watered environments (Li and Wang, 2023), with different varieties exhibiting varying water demands across ecologies (irrigated lowland, rainfed lowland, rainfed upland). Drought remains one of the most severe constraints to rice growth and productivity capable of causing yield losses of up to 100%, particularly when stress coincides with sensitive developmental stages (Ahmadikhah and Marufinia, 2016; Hussain et al., 2022). Future projections suggest that by 2050, climate change-driven events like drought and floods may render most arable lands less productive, potentially costing most African nations more than €47 billion/year, and directly impacting the livelihood of rainfed rice farmers (WMO, 2022). Thus, improving drought resilience in rice is a pressing challenge for sustainable agriculture (Darko et al., 2025). To address these challenges, breeding program such as Africa Rice Center (AfricaRice) development of NERICA (New Rice for Africa) varieties have made important progress in improving drought tolerance, particularly at the flowering stage. These efforts have proven instrumental in improving livelihoods and food security in several African countries (Arouna et al., 2017). Nevertheless, current breeding strategies have focused on adult or reproductive stages, often overlooking drought responses during the early vegetative phase. This is a critical gap, as young seedlings are especially vulnerable to water deficit, and early-stage stress can profoundly affect subsequent growth and yield potential.

When drought occurs, plants activate a complex suite of adaptive responses, including morphological, biochemical, physiological, and molecular changes (Movahedi et al., 2023). These responses involve stomatal closure, altered root development, accumulation of protective molecules such as phytohormones (e.g., abscisic acid, jasmonic acid, gibberellin, auxin), osmoprotectants such as proline, and stress response proteins such as dehydrins and and LEA (Late Embryogenesis Abundant) proteins (D’Ippólito et al., 2021; Aslam et al., 2022; Movahedi et al., 2023). Drought stress also triggers the accumulation of plant proteases, protease inhibitors, as well as stay-green proteins (Kamal et al., 2019; Kumar et al., 2022; Moloi and Ngara, 2023). Another layer of drought-induced molecular reprogramming involves the induction of various transcription factors (e.g., *bZIP*, *WRKY*), signalling pathways (e.g., *Ca2+*, *MAPKs*), and regulatory gene networks that orchestrate cellular adaptation (Aslam et al., 2022).

Advances in transcriptomics have enabled detailed dissection of these adaptive responses, revealing that drought-responsive gene expression varies not only by genotype but also by developmental stage and tissue type (Huang et al., 2014, Huang et al., 2021; Y. Zhang et al., 2016; Yang et al., 2019; Tiwari et al., 2021; Baldoni et al., 2021; Kaur et al., 2023; Park and Jeong, 2023);. Such insights can inform the design of molecular breeding strategies aimed at improving drought resilience from the seedling stage onward.

In previous field screening studies at the CSIR-Crops Research Institute, four elite rice varieties (APO, CRI-Enapa, ART132-35-1-1-B-B and CRI-Amankwatia) were identified as either drought tolerant (APO and CRI-Enapa) or drought sensitive (ART132-35-1-1-B-B, CRI-Amankwatia) at later growth stages (Adjah et al., 2024, Adjah et al., 2025). However, with the increased frequency of dry spells in sub-Saharan Africa, it is critical to understand how these elite varieties respond to drought at early vegetative stages, which are often overlooked in conventional selection protocols.

In this study, we designed a protocol to mimic drought conditions in young rice plants grown under controlled laboratory conditions and evaluated both phenotypic (root and shoot biomass) and transcriptomic responses among the four varieties. Comparative transcriptome analysis, as well as co-expression network analysis, focused on APO and CRI-Enapa (tolerant) and ART132-35-1-1-B-B (sensitive), leading to the identification of differentially expressed genes (DEGs) and high regulatory hub genes that are associated with early-stage drought tolerance. These findings provide valuable molecular markers and regulatory networks for breeding programs targeting drought tolerance from the seedling stage.

## 2 Materials and methods

### 2.1 Background information of the rice genotypes evaluated under field drought conditions

Adjah et al. (2025) comprehensively describes the genetic background of the genotypes [APO, CRI-Enapa (from now on described as Enapa), CRI-Amankwatia and ART132-35-1-1-B-B (from now described as ART32)] used in this study. Basically, these genotypes are a subset of 100 that were initially evaluated for tolerance to drought stress under field conditions at the CSIR-Crops Research Institute, Kumasi, Ghana, during the 2021–2022 dry season where APO, CRI-Enapa were identified as tolerant while ART32 and CRI-Amankwatia were sensitive to drought stress (**Supplementary File 1_S1**).

### 2.2 Evaluation of the rice genotypes grown in pots under controlled drought conditions

Three seedlings of seven-days old of each of the four cultivars (APO, CRI-Enapa, ART32 and CRI-Amankwatia) were transplanted into 500 ml transparent pots prefilled with 400g of compost laid in completely randomised block design with three replications for two experimental setups; control where plants received continuous supply of water throughout the experiment and stress where water was withheld from 10-day old seedlings and lasted for 30 days until the pots reached 50% of water content. Both control and stress setups were maintained under 12 h light/12 h dark at 28°C. Pots in the stress setup are weighted every two days to track the water loss, while the pots under control conditions were topped up with the water lost at each measurement. We selected 50% soil water content as the drought threshold because this level corresponds to a physiologically meaningful drought stress response in rice. Previous studies have successfully used 50% field capacity to screen rice genotypes under controlled pot conditions, demonstrating that this moisture level induces clear drought responses while avoiding lethal stress (Lone et al., 2019; Wang et al., 2021). Data were collected on shoot fresh weight and root fresh weight.

### 2.3 RNA extraction

RNA was extracted from APO, CRI-Enapa, ART32 and CRI-Amankwatia. However, only three cultivars (APO, CRI-Enapa, and ART32) were used for the RNA sequencing (RNA-seq) analysis. For the quantitative real-time reverse transcription PCR (RT-qPCR) validation, all four cultivars (APO, CRI-Enapa, ART32, and CRI-Amankwatia) were included. The additional cultivar, CRI-Amankwatia, was incorporated in the RT-qPCR analysis to independently validate the RNA-seq results in a genotype that was not part of the original RNA-seq experiment. For the extraction protocol shoots and roots from both control and stress setups were sampled at ZT4-5 (ZT, Zeitgeber time, time after lights on) in liquid nitrogen and stored at -80°C until RNA extraction. Three biological replicates were used for RNA extraction; each included a pool of six individual plants. Total RNA was isolated using the RNeasy^®^ kit (QIAGEN GmbH, Germany) and treated with DNase I to eliminate any contaminating DNA. The RNA concentration was initially measured using a NanoDrop 2000 spectrophotometer (Thermo Fisher Scientific, Waltham, MA, USA; ND-2000), and then its quality and concentration further assessed using Qubit. Only high-quality total RNA (1 µg) (OD^260/280^ = ~2.0, OD^260/230^ ≥ 2.0, RIN ≥ 6.0, 28S:18S ≥ 1.0) was used as input material to construct the RNA-seq library. Integrity/degradation of RNAs was then checked on 1.5% agarose gel, and on the Agilent 4200 TapeStation System (^©^ Agilent Technologies, Inc. 2020, USA) according to the manufacturer’s protocol.

### 2.4 RNA sequencing

A total of 18 RNA-seq libraries representing three distinct biological replicates were prepared from shoots collected from three rice cultivars (APO, Enapa and ART32) grown under control and stress conditions. Illumina sequencing analysis was prepared by *Sequentia Biotech* (Barcelona) using an Illumina Novaseq 6000 platform with read length of 150nt and a PE (paired-end) library type.

To ensure high-quality data for subsequent analysis, the RNA-seq libraries underwent preprocessing using Trimmomatic (Bolger et al., 2014) to clean Illumina adapters sequences. This involved utilizing the Trimmomatic tool to trim reads while ensuring a minimum sequencing quality threshold of 20. Mapping was performed using STAR (Dobin and Gingeras, 2015) with default parameters and reference genome utilizing the previously assembled and annotated genome (ASM465v1) from the rice plant *Oryza sativa indica* from *EnsemblPlants*. Count matrix was built using *featureCounts*; only counting reads with a minimum alignment quality of 30. Once the count matrix was created, counts were filtered using a threshold of 10 counts per feature (row-wise), ensuring that only features with a minimum level of expression were retained. Furthermore, features were required to have counts in at least two samples to be included in the final analysis. This filtering step helped to focus on robustly expressed features across multiple samples, enhancing the reliability of downstream analyses.

### 2.5 Transcriptomic and differential expression analysis

Differential expression analysis was conducted using the *edgeR* tool (Empirical Analysis of Digital Gene Expression in R package). In this analysis, we focused on identifying differentially expressed genes (DEGs) with a False Discovery Rate (FDR) of less than 0.05. Three different comparisons were analysed: Drought-stress (APO) vs Control (APO), Drought-stress (Enapa) vs Control (Enapa), and Drought-stress (ART32) vs Control (ART32). Control samples are used as reference for the fold change (FC) calculation using the log_2_FC. Before applying edgeR, a filtering step was implemented to refine the RNA-seq dataset. By filtering out these genes, the overall quality of the dataset was improved. In all cases, unless otherwise indicated, the count matrix was filtered using a filter of 10 counts (row-wise) and counts in at least two samples, and differentially expressed (DE) regulators (RE) or Genes for tolerant plants were defined using a False Discovery Rate (FDR) < 0.05, with a difference in abundance with Log_2_FC ≥ 2 upregulated and Log_2_FC ≤ -2 downregulated, at a significance level ≤ 0.0001. The *HTSFilter* function was used to remove lowly expressed genes that might have added noise to the differential expression analysis. The *HTSFilter* algorithm calculates a Global Jaccard similarity index (on the Y-axis) between the samples for each condition based on different normalized minimum read counts (on the X-axis). A volcano plot was generated to visualize the differential gene expression between conditions. SRplot was used to generate the Venn diagram (Tang et al., 2023).

Finally, a GO enrichment analysis of upregulated and downregulated genes was performed using the *EnsemblPlants* database by comparing with Arabidopsis (*Arabidopsis thaliana*, TAIR10) or rice (*Oryza sativa indica*, ASM465v1) model plants. Bubbleplots were generated to visualize the results using ggplot2 package in R version 4.5.1. These plots were separated by ontology categories (biological process, molecular function, and cellular component), allowing for a comprehensive exploration of enriched GO terms associated with the identified DEGs.

### 2.6 Co-expression network analysis and key regulatory hubs identification

Gene expressions count data derived from RNA-sequencing and all subsequent analysis were done using R version 4.5.1. Raw counts were first processed and normalized using DESeq2 package (Love et al., 2014) to ensure accurate comparison of gene expression levels across samples. Genes with very low expression (total read counts <10 across all samples) were removed to reduce background noise. Variance stabilizing transformation was then applied to normalize the data and generate a stabilized expression matrix suitable for downstream network analysis. To reduce computational complexity while focusing on the most informative genes, a variance-based filtering step was performed, and the top 20% most variable genes were retained for subsequent analyses (**Supplementary Additional File 5**).

Gene co-expression networks were constructed using the WGCNA package (Langfelder and Horvath, 2008). A soft-thresholding power was selected based on the scale-free topology criterion, and modules of co-expressed genes were identified using hierarchical clustering with a minimum module size of 30 genes and a module merging threshold of 0.25. Module eigengenes were calculated to summarize the overall expression pattern of each module, and Pearson correlation analysis was used to assess the relationships between module eigengenes and the experimental traits including shoot fresh weight and root fresh weight under control and drought stress. Modules showing significant correlations with the traits (p < 0.05) were considered biologically relevant and selected for further analysis. Hub genes within these modules were identified based on gene significance (GS), which is the correlation between gene expressions and traits, and module membership (kME), which is the correlation between gene expression and the corresponding module eigengene.

To further explore regulatory relationships among genes, a gene regulatory network was inferred using mutual information-based approaches implemented in minet package (Meyer et al., 2008). Regulatory interactions were predicted using the CLR algorithms. To simplify the network and highlight the most robust regulatory interactions, only the top 5% strongest edges were retained for visualization and hub gene detection. Functional characterization of the identified hub genes was performed through GO enrichment analysis using the R package gprofiler2 (Peterson et al., 2020) with annotations retrieved from *EnsemblPlants*. Enriched biological processes were interpreted to identify pathways associated with drought response. Network visualization and hub connectivity analysis were performed using igraph and ggraph packages from R version 4.5.1, where node size reflected gene connectivity and edges represented predicted regulatory interactions.

### 2.7 Quantitative real-time reverse transcription-PCR analysis

All analyses were performed on two biological replicates and three technical replicates per sample, collected as described for the RNA-Seq experiment. cDNA synthesis was performed using NZY First-Strand cDNA Synthesis Kit, separate oligos (NZYtech, Lisbon, Portugal) according to the manufacturer’s protocol. The *RT-qPCR* was used to verify a subset of selected known drought genes involved in drought response mechanisms, using Takara SYBR Green Taq Polymerase according to the manufacturer’s protocol. The primers of the genes used were retrieved from Kaur et al. (2023); Park and Jeong (2023); Zhang et al. (2016) and Huang et al. (2014), (**Supplementary Additional File 1_S2**). Three rice genes with various function categories were selected and tested in 20 μL reactions using the SYBR^®^ Green PCR Master Mix kit (Applied Biosystems, CA, USA), following the manufacturer’s protocol, using an ABI Prism 7900 Sequence Detection System (Applied Biosystems). The relative expression of each gene was calculated according to the method of 2^-△Ct^ (Udvardi et al., 2008). The *Actin 1* gene (*LOC_Os03g50885*) was used as endogenous references for *RT-qPCR*.

### 2.8 Statistical analysis

Two-way analysis of variance (ANOVA) for each trait was done using the GLM procedure of the Statistical Analysis System (SAS) version 9.4 for Windows. To ensure normal distribution of the traits, before the ANOVA, all the traits were z-score transformed. Duncan’s multiple rank test was used to separate the means among the genotypes screened in the drought stress experiments upon the significance of the ANOVA. Graphs were made using R software version 4.5.1.

## 3 Results

### 3.1 Evaluation of rice genotypes grown under controlled and drought conditions

**Figure 1:**
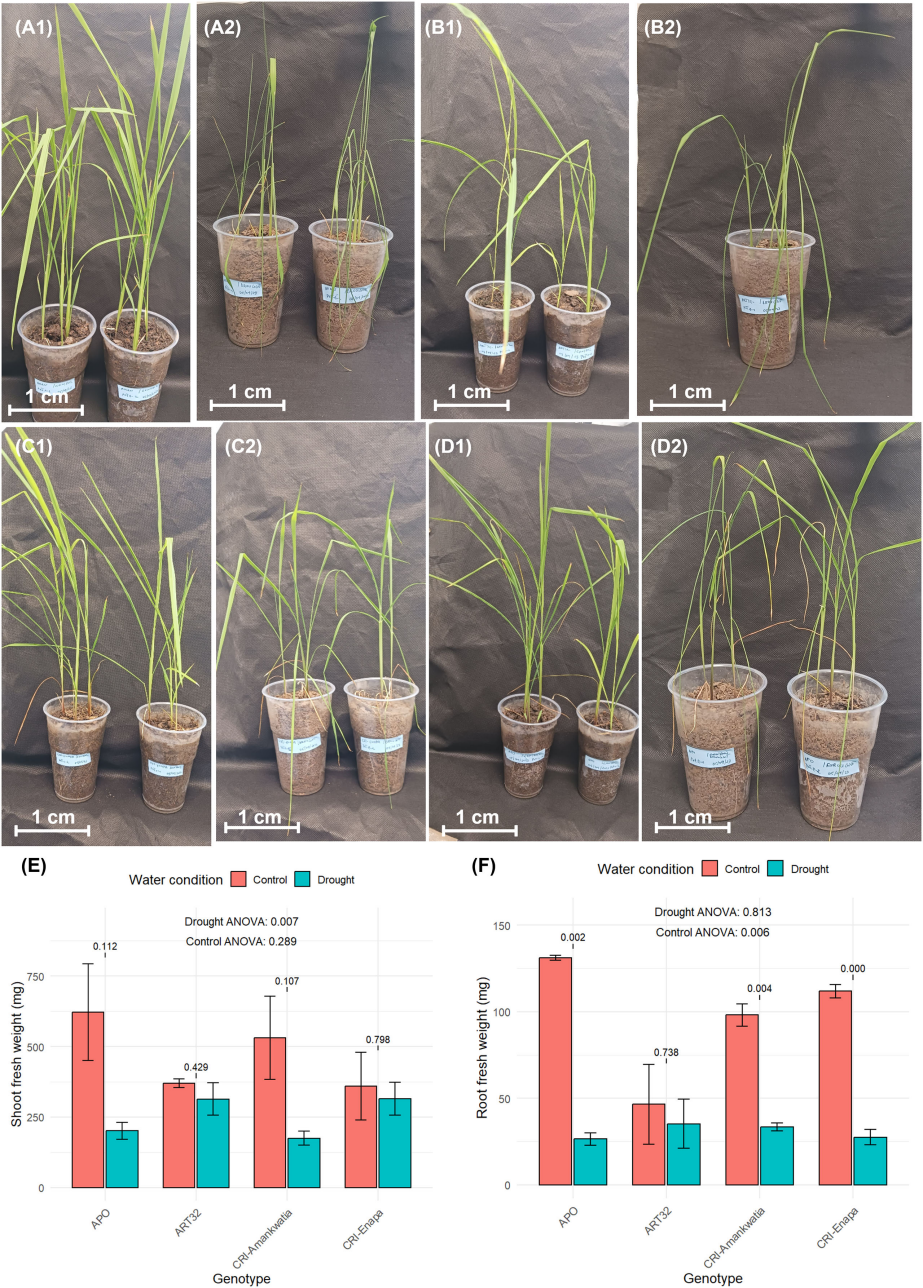
Performance of the four rice genotypes for shoot fresh weight and root fresh weight evaluated under control and drought conditions. Representative photos of 40-day old plants, grown under control and drought conditions. (A1) CRI-Amankwatia under control conditions, (A2) CRI-Amankwatia under drought-stress; (B1) ART32 under control conditions, (B2) ART32 under drought-stress, (C1) Enapa under control conditions, (C2) Enapa under drought-stress (D1) APO under control conditions, (D2) APO under drought-stress. **(E)** Shoot fresh weight of all the tested varieties grown under control and drought conditions. **(F)** Root fresh weight of all the tested varieties grown under control and drought conditions. Data are means (± SE) of three biological replicates (n=6). Relative shoot or root fresh weight is estimated as the ratio of shoot or root fresh weight value under drought-stress on shoot or root fresh weight value under control conditions. Paired t-tests numeric p-values using brackets were used to assess the significance differences between control vs drought for each genotype. Statistically significant differences among the genotypes under control or drought are indicated with their corresponding *p-value* at the top of each figure as determined by two-way ANOVA followed by Duncan test. Statistically significant at 5% level of significance. Scale bar corresponds to 1cm.

Ten-day old seedlings were exposed to 30 days of drought-stress until their pots reached 50% of water content. At this point physiological parameters were assessed (e.g., root and shoot fresh weight) for all the tested varieties (APO, Enapa, ART32 and CRI-Amankwatia). Shoot fresh weight data showed that under control conditions APO had the highest fresh weight (622.04 ± 146.93 mg), whereas Enapa (359.21 ± 171.62 mg) recorded the lowest (**Figure 1**). Under drought conditions, however, Enapa displayed the highest shoot fresh weight (315.43 ± 30.58 mg), whereas CRI-AMANKWATIA had the lowest (174.55 ± 58.33 mg). When the relative shoot fresh weight was analysed, Enapa and ART32 displayed the higher values (**Figure 1**).

Similarly, APO plants recorded the highest root fresh weight under control conditions (131.26 ± 11.22 mg), while ART32 recorded the lowest (46.49 ± 23.05 mg). Under drought conditions, however, ART32 displayed the highest root fresh weight (35.27 ± 14.26 mg), and APO recorded the lowest (26.43 ± 3.90 mg). ART32 had the higher relative root fresh weight, whereas Enapa and APO had the lowest (**Figure 1**).

### 3.2 Whole transcriptional changes across the three genotypes evaluated

**Figure 2:**
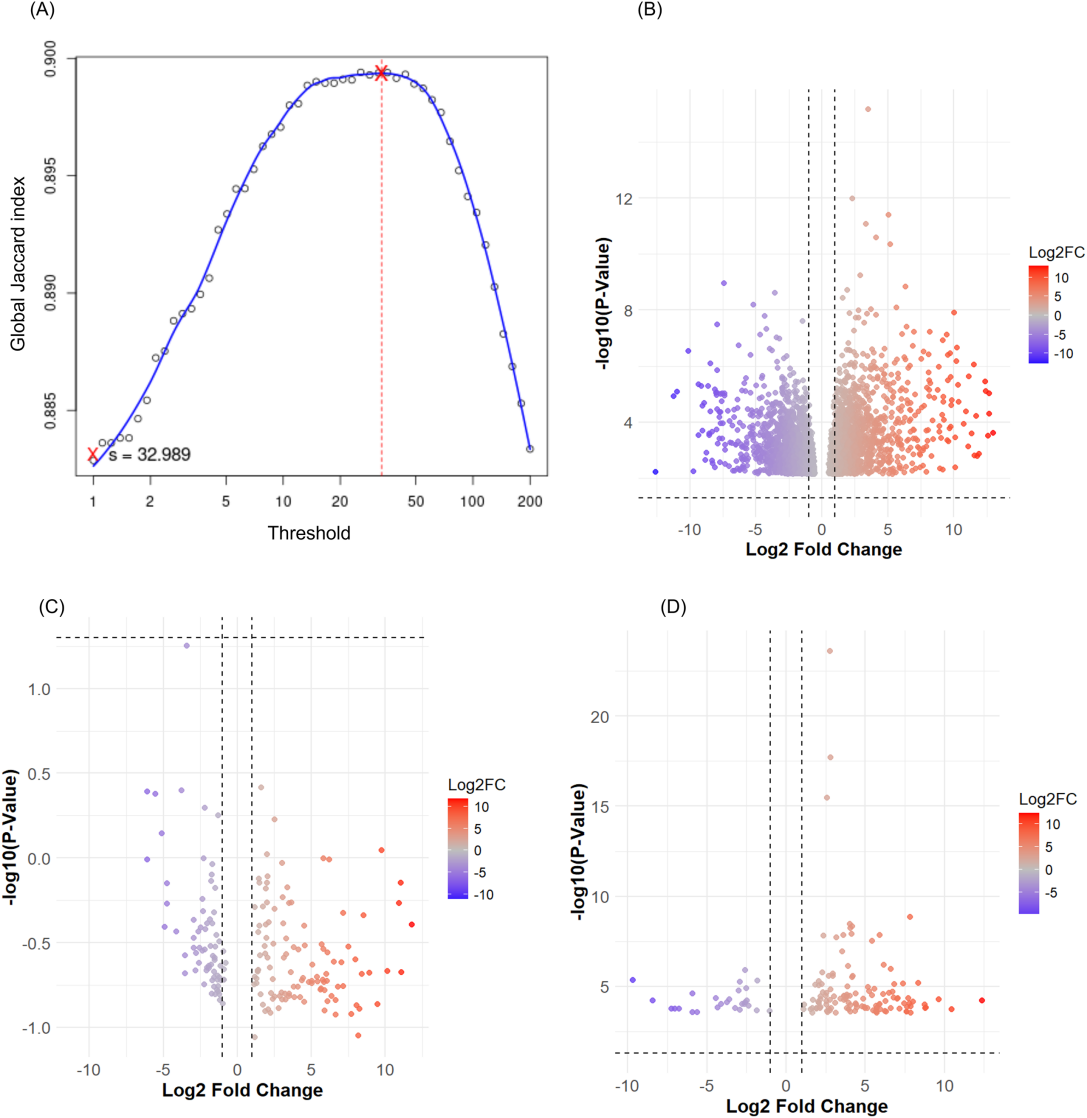
Global Jaccard similarity index between the samples for each condition based on different normalized minimum read counts **(A)**, and distribution of differentially expressed genes between control and drought conditions using volcano plot [**(B)** (APO), **(C)** (Enapa) and **(D)** (ATR32)] among the three rice varieties tested. On **(B–D)**, each point represents a comparison of a single feature (gene expression) between conditions. Blue points represent downregulated genes (negative Log2FC) and are significantly different from the control conditions with a high degree of confidence. Red points represent upregulated genes (positive Log2FC) and are significantly different from the control. Grey points are near to zero with no significant change.

RNA concentration of all the samples is presented in **Supplementary Additional File 1_S3**, and the RNA integrity/degradation analysis was done on a 1.5% agarose gel which confirmed the absence of degradation (**Supplementary Additional File 1_S4**). RNA-Seq analysis revealed a total input of 30.1 to 44.01 million reads that were mapped for each sample, among which the uniquely mapped reads ratio varied between 89.93%-92.56%; whereas 2.78% to 4.15% of the reads were mapped to multiple loci, and 4.64% to 6.08% of the reads were unmapped to the reference genome (**Supplementary Additional_S5**). The unique matching reads were used for further analysis. The Global Jaccard similarity index (on the Y-axis) between the samples for each condition based on different normalized minimum read counts (on the X-axis) showed that for s = 32.989, the replicates had the highest similarity; therefore, this value was used as the threshold (**Figure 2A**). In the eighteen libraries prepared 42031 genes (transcripts) were identified, of which 16809 were classified as gene outliers using *edgeR* leaving 25222 filtered genes.

### 3.3 Comparative transcriptome profiling of three genotypes under drought-stress

**Figure 3:**
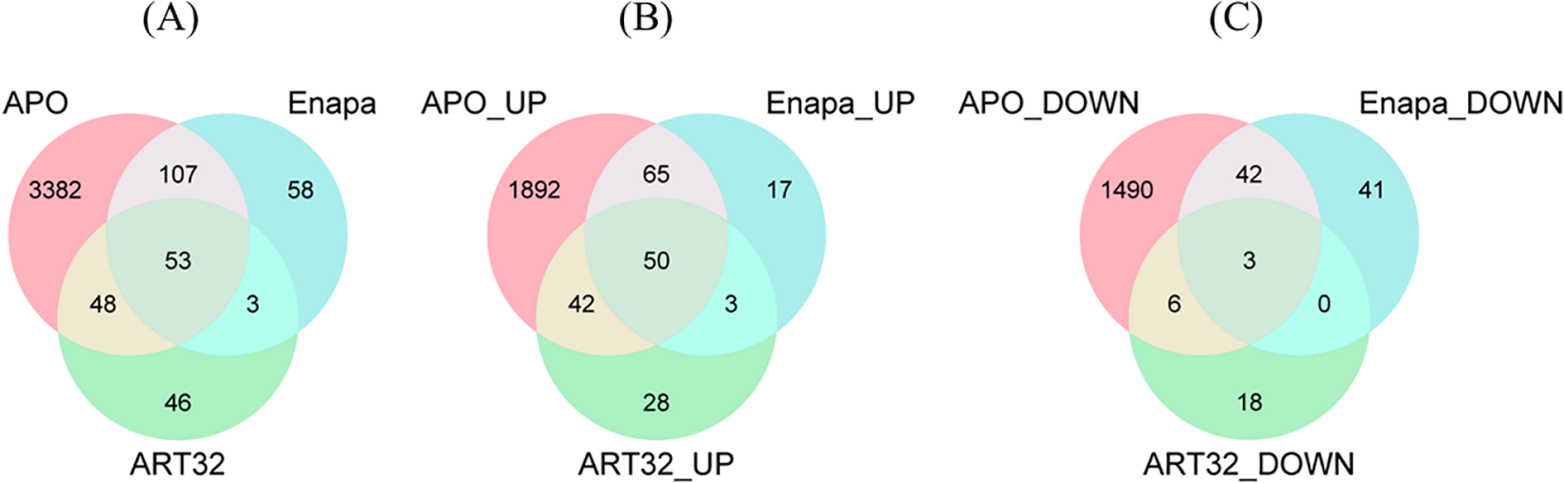
Venn diagram of differentially expressed genes (DEGs) in APO, Enapa, and ART32 evaluated under early vegetative-stage control and drought conditions among three rice varieties. Analysis of total **(A)**, upregulated **(B)**, and downregulated **(C)** DEGs identified with adjusted *p-value* < 0.001.

To determine the differential expression in the drought-induced transcriptomes in the three genotypes, the changes in transcript abundance in the three genotypes under drought-stress compared with their corresponding control values were analysed. In APO, a total of 3590 DEGs were identified, of which 2049 (57%) were upregulated genes (Log_2_FC > +2, FDR ≤ 0.05) and 1541 (43%) were downregulated (Log_2_FC > -2, FDR ≤ 0.05). In Enapa, a total of 221 DEGs were identified and 135 (61%) of these were upregulated (Log_2_FC > +2, FDR ≤ 0.05), while 86 (39%) genes were downregulated (Log_2_FC > -2, FDR ≤ 0.05). In ART32, a total of 150 DEGs were identified, of those, 123 (82%) transcripts were upregulated (Log_2_FC > +2, FDR ≤ 0.05), while 27 (18%) were downregulated (Log_2_FC > -2, FDR ≤ 0.05). A volcano plot was used to visualize the DEGs identified in control and drought conditions in the three varieties (**Figures 2B–D**). When these DEGs were compared among the three tested varieties, we found that 53 were commonly regulated (**Figure 3A**) with 50 (94%) showing upregulation (**Figure 3B**) and only 3 (6%) were downregulated (**Figure 3C**). Detailed analysis of the commonly regulated DEGs in APO and ENAPA revealed 107 transcripts, where 65 (61%) were upregulated and 42 (39%) downregulated. Moreover, drought-tolerant APO and drought-sensitive ART32 shared 48 DEGs, 42 (87.5%) of those were upregulated and only 6 (12.5%) were downregulated. On the other hand, when drought-tolerant ENAPA and drought-sensitive ART32 were compared, only 3 commonly regulated DEGs were identified and all showed upregulation (**Figure 3**).

Our analysis identified 107 commonly regulated DEGs between APO and Enapa, whereas 3382 and 58 APO-specific and Enapa-specific transcripts were recorded, respectively. ART32 displayed smaller transcriptional changes with only 46 uniquely regulated associated transcripts.

### 3.4 Comparative gene ontology enrichment analysis of three varieties under control and drought conditions

Gene ontology analysis allowed the characterization of 21.72% of the DEGs in APO, 14.93% in Enapa and 12.67% in ART32. The main functional categories identified were: phytohormones, stress response, transcription regulation, ROS homeostasis, signalling transduction, carbohydrate metabolism and osmotic adjustment, and plant proteases and protease inhibitors.

#### 3.4.1 Common DEGs in APO, ENAPA and ART32

GO analysis of the 53 common DEGs in the three varieties highlighted, within the phytohormone related transcripts, *ABA receptor 6* (*BGIOSGA010919*) and *Gibberellin-induced A20/AN1 zinc-finger protein* (*BGIOSGA028955*) which were downregulated in all the genotypes. Within ‘stress responses’ dehydrins from *Group 2 late embryogenesis abundant* (*LEA*) *proteins*, *Dehydrin Rab16C* (*BGIOSGA034053*) and *Group 3 LEA protein* (*BGIOSGA000945)* were identified as upregulated in the three varieties. ‘Carbohydrate metabolism’-related transcripts such as the *bidirectional sugar transporter SWEET15 (BGIOSGA006395)* were also upregulated in all the three varieties. Similarly, among the protein kinases (signal transduction), *phosphoglycerate kinase (BGIOSGA020715)* was also upregulated in all the varieties (**Supplementary Additional Files 2–4**).

#### 3.4.2 Gene ontology enrichment of commonly expressed DEGs in APO and ENAPA

We searched for commonly upregulated transcripts in the two ‘drought-tolerant’ varieties APO and Enapa and identified that *Dehydrin Rab16C* (*BGIOSGA034053*) and *Dehydrin Rab16D* (*BGIOSGA034054*) were upregulated in both. We also identified *dehydration-responsive element binding protein 1 (DREB1)* isoforms which were downregulated in APO and ENAPA including *DREB1E (BGIOSGA016950), DREB1A (BGIOSGA029415), DREB1H (BGIOSGA029416) and DREB1B (BGIOSGA029417).* Transcription factors *WRKY TF 67 (BGIOSGA018676) and WRKY TF 53 (BGIOSGA019646)* were significantly downregulated in APO and ENAPA, whereas *similar to Heat stress transcription factor B-2b (BGIOSGA026537)* was upregulated in both genotypes under drought. Among the protein kinase group, only *Non-specific serine/threonine protein kinase (BGIOSGA028744)* was differentially expressed and upregulated in both APO and ENAPA (**Supplementary Additional Files 2, 3**).

#### 3.4.3 Gene ontology enrichment of commonly expressed DEGs in Enapa and ART32

GO analysis of the three upregulated common DEGs in Enapa and ART32 included *BGIOSGA019996*, *BGIOSGA007111*, *BGIOSGA004381*, although none of them was characterized (**Supplementary Additional Files 3, 4**).

#### 3.4.4 Gene ontology enrichment of commonly expressed DEGs in APO and ART32

GO analysis of the common DEGs in APO and ART32 highlighted those involved in carbohydrate metabolism and osmotic adjustment including *similar to ATCAX5 calcium:cation antiporter/cation:cation antiporter* (*BGIOSGA007496*), *Probable esterase PIR7A* (*BGIOSGA000172*), *Isoamylase* (*BGIOSGA026650*) and *Glucose-1-phosphate adenylyltransferase* (*BGIOSGA017490*) which were all upregulated. The six downregulated DEGs including *BGIOSGA003364*, *BGIOSGA005185*, *BGIOSGA005899*, *BGIOSGA024147*, *BGIOSGA024735* and *BGIOSGA030196* were uncharacterised (**Supplementary Additional Files 2, 4**).

#### 3.4.5 Gene ontology enrichment of uniquely expressed DEGs in ART32 under control and drought conditions

**Figure 4:**
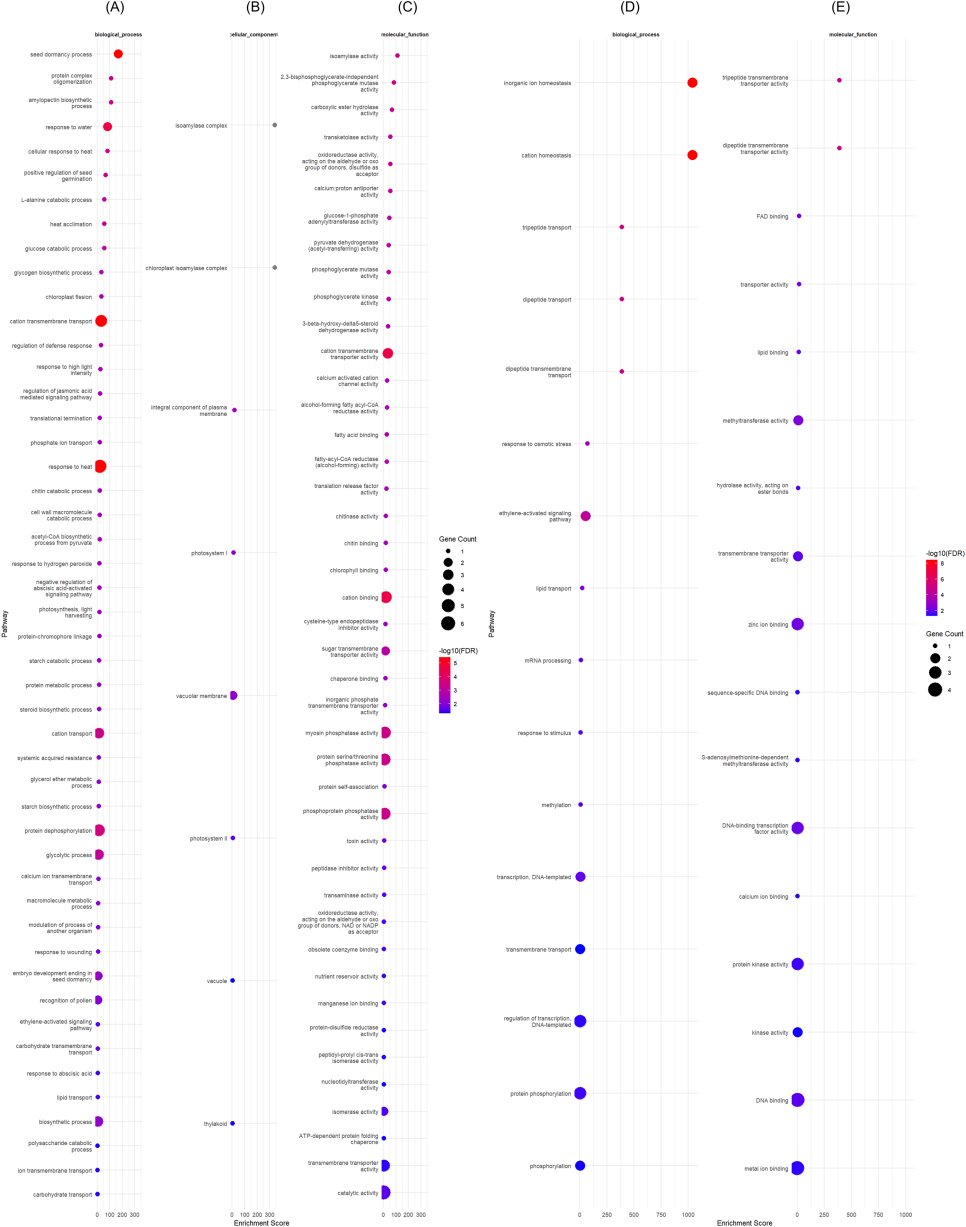
Gene ontology of differentially expressed genes (DEGs) in rice variety ART32 grown under control and drought conditions at early vegetative stage. **(A–C)** show the gene ontology (GO) results for upregulated DEGs while **(D, E)** for downregulated DEGs, respectively. DEGs were identified with adjusted *p-value* < 0.001.

We then investigated which GO categories would be specifically enriched in each variety. In ART32 we found that *Fatty acyl-CoA reductase* was upregulated, as well as *cysteine proteinase inhibitor* (*BGIOSGA030147*), while *Probable plastid-lipid-associated protein 14, chloroplastic* (*BGIOSGA004137*), *BGIOSGA008436* and *BGIOSGA012597* were downregulated (**Figure 4**).

#### 3.4.6 Gene ontology enrichment of uniquely expressed DEGs in Enapa under control and drought conditions

**Figure 5:**
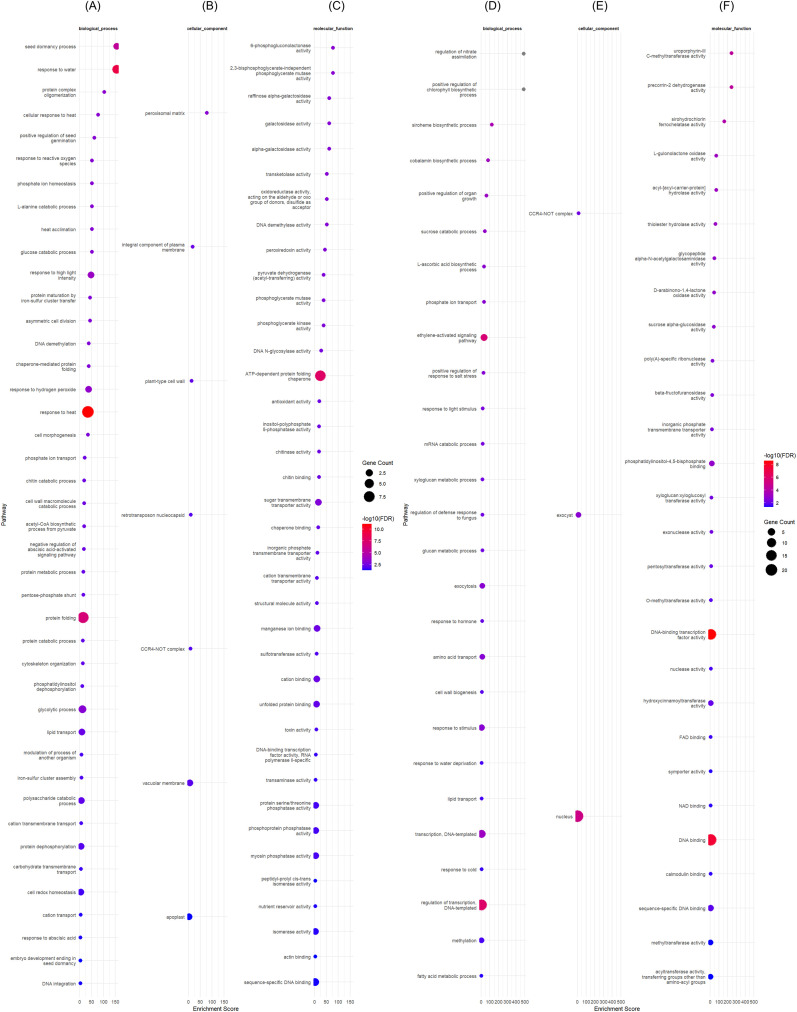
Gene ontology of differentially expressed genes (DEGs) in rice variety Enapa grown under control and drought conditions at early vegetative stage. **(A–C)** show the gene ontology (GO) results for upregulated DEGs while **(D–F)** for downregulated DEGs, respectively. DEGs were identified with adjusted *p-value* < 0.001.

Among the characterized DEGs downregulated in ENAPA, we identified the transcription factor (TF) *Ethylene-responsive TF 6 (BGIOSGA033447)* and Acyl-hydrolase (*BGIOSGA005944*), while all upregulated DEGs (including *BGIOSGA036324*, *BGIOSGA018976* and *BGIOSGA023737*) were uncharacterized (**Figure 5**).

#### 3.4.7 Gene ontology enrichment of uniquely expressed DEGs in APO

Within the GO enrichment analysis for ‘phytohormone related proteins’, we identified 12 *Auxin-responsive* genes which were downregulated in APO (**Supplementary Additional File 2**). We found that three cytokinin (CK) related genes (*BGIOSGA008655, BGIOSGA011686, BGIOSGA016764*) were also repressed in APO, except *cytokinin dehydrogenase 8* (*BGIOSGA016782*) which was up-regulated (**Supplementary Additional File 2**). Another group of APO-specific DEGs included five ABA/stress-induced transcripts (*HVA22-like proteins*) of which three (*BGIOSGA000885, BGIOSGA032228 and BGIOSGA033727*) were down-regulated, while two (*BGIOSGA035307 and BGIOSGA000885*) were up-regulated (**Supplementary Additional File 2**). There were also several phytohormone-related transcripts uniquely regulated in APO, such as ABA *receptor 9* (*BGIOSGA033490*), *Ethylene receptor-like protein 1* (*BGIOSGA015886*) and *Protein BRASSINOSTEROID INSENSITIVE 1* (*BGIOSGA000907*) that were all downregulated (**Supplementary Additional File 2**). All six jasmonate (JA) biosynthesis-related DEGs, encoding two *lipoxygenase* (LOX), four *3-ketoacyl-CoA synthase* were downregulated, while *Acyl-coenzyme A oxidase* was upregulated in APO under drought conditions (**Supplementary Additional File 2**).

Among the characterised DEGs, 13 redox regulation related genes were differently expressed in APO compared to ENAPA and ART32 under drought (**Supplementary Additional Files 2–4**). These transcripts encoded *glutathione peroxidase* (*BGIOSGA034146*), *Electron transfer flavoprotein-ubiquinone oxidoreductase* (*BGIOSGA033279*), *FAD-dependent oxidoreductase family protein* (*BGIOSGA040585*), and *glutathione S-transferase* (*BGIOSGA034146, BGIOSGA009694*) all of which upregulated under drought (**Supplementary Additional Files 2–4**). *Proline aminopeptidase* (*BGIOSGA017731*) was uniquely upregulated in APO while *group 3 LEA protein* (*BGIOSGA020289*) and *STAY-GREEN protein (BGIOSGA029383)* were upregulated transcripts in APO (**Supplementary Additional File 2**).

The characterised DEGs from the ‘carbohydrate metabolism and osmotic adjustment’ categories included *Glycosyltransferase* and *UDP-glucosyl transferase (UGT) family proteins* and *Ca2+-permeable mechanosensitive channel protein*. In addition, within the ‘metabolism’ category we identified the *Bidirectional sugar transporter SWEET Gene Family*, including *SWEET6b (BGIOSGA003948)* and *SWEET6a (BGIOSGA003951)* which were upregulated. However, *SWEET11 (BGIOSGA026582)* and *SWEET14 (BGIOSGA033930)* were downregulated (**Supplementary Additional File 2**). The massive transcriptional reprogramming in APO also included several families of transcription factors (TFs), including *bZIP TFs* (*BGIOSGA000430, BGIOSGA004837, BGIOSGA005551, BGIOSGA011218* and *BGIOSGA012577*), *transcription initiation factor TFIID subunit 15b* (*BGIOSGA002844*), *TF Dehydration and salt stress tolerance* (*BGIOSGA002846*), *GATA transcription factor 1 (BGIOSGA011849), Ethylene-responsive TF ERF061 (BGIOSGA017525)*, and *WRKY TF 47 (BGIOSGA023706)*, which were significantly upregulated under drought. On the other hand, we also identified downregulated TFs such as *TF B3 family protein/auxin-responsive factor AUX/IAA-related (BGIOSGA014158)* and *Ethylene-responsive TF 6 (BGIOSGA033447)*. Other upregulated transcripts unique to APO included *similar to Heat stress TF Spl7 (BGIOSGA016941)*, and *similar to Heat stress transcription factor Spl7 (BGIOSGA030901)* (**Supplementary Additional File 2**).

The protein kinase transcripts which were differentially expressed in APO under drought conditions included *serine/threonine protein kinase related genes*, and *receptor kinases (RKs)* such as the *Brassinosteroid (BR) receptor kinase (BGIOSGA027144)*, and the *LRR receptor kinase BAK1 (BGIOSGA028076)* (**Supplementary Additional File 2**). Interestingly, *calcium-dependent protein kinase 29 (CDPK 29, BGIOSGA014554)* and *Diacylglycerol kinase (BGIOSGA017202 and BGIOSGA027553)* were both repressed in APO under drought conditions.

**Figure 6:**
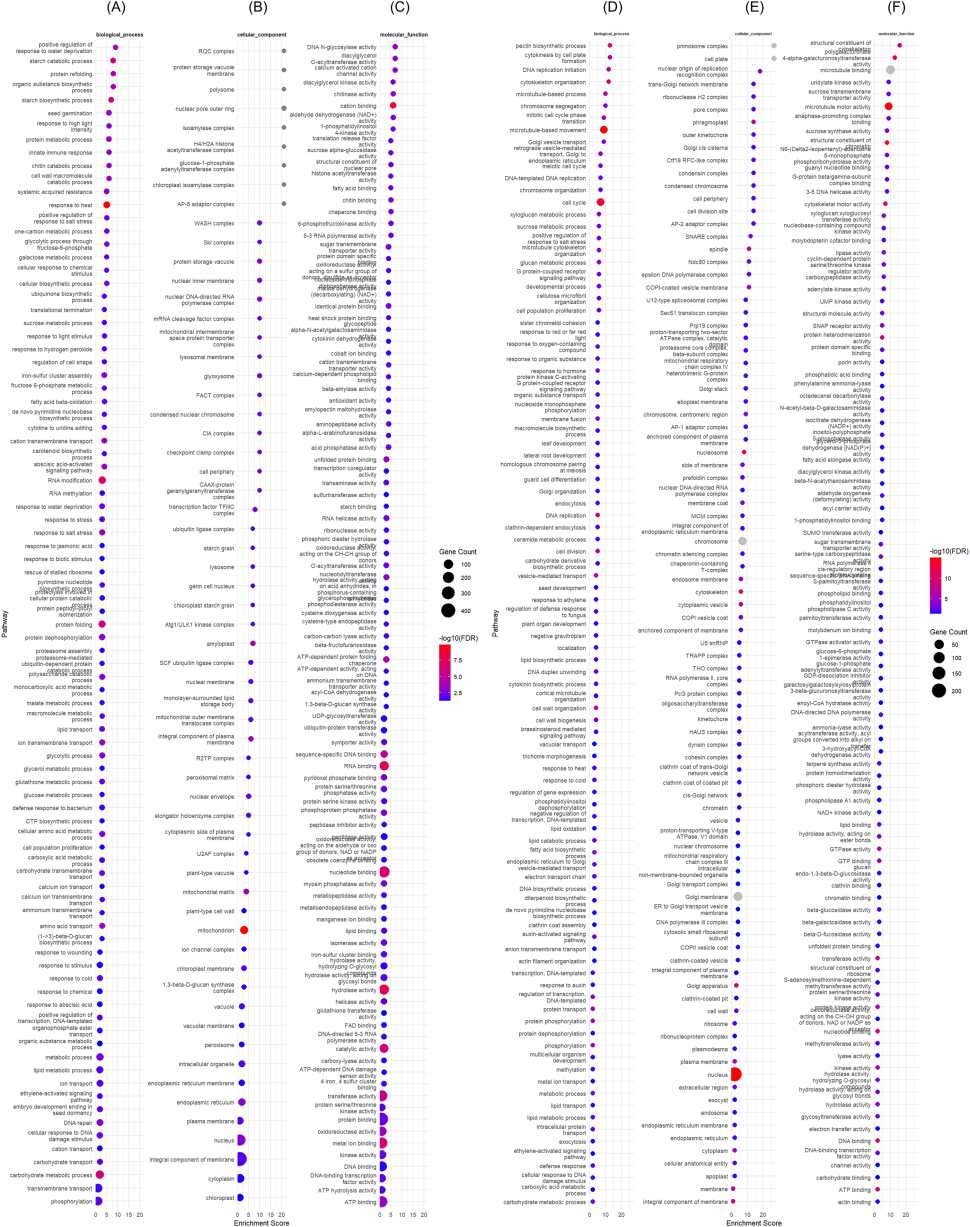
Gene ontology of differentially expressed genes (DEGs) in rice variety APO evaluated under early vegetative stage drought and control conditions. **(A–C)** show the gene ontology (GO) results for upregulated DEGs while **(D–F)** for downregulated DEGs, respectively. DEGs were identified with adjusted *p-value* < 0.001.

We identified a total of 19 genes related to plant proteases and protease inhibitors that were unique APO DEGs. These genes included *carboxypeptidases*, *serine hydroxy methyltransferase (BGIOSGA018007 and BGIOSGA022876)*, *senescence-specific cysteine protease SAG39 (BGIOSGA015901)*, *cysteine proteinase inhibitor* (*BGIOSGA017804*), *plant cysteine oxidase 3 (BGIOSGA027939)*, and *ATP-dependent zinc metalloprotease FTSH 6, chloroplastic (BGIOSGA021611)* (**Figure 6**).

Based on the differential expression analysis and the GO enrichment categories, most of the drought regulated DEGs were identified in APO, followed by Enapa, and only a reduced number of DEGs were uniquely expressed in ART32.

Considering that plants will respond to drought-stress by activating ABA signalling, we then investigated how transcripts associated with this pathway were regulated in the three varieties tested.

### 3.5 Regulation of ABA signalling at early vegetative stages of rice varieties grown under control and drought conditions

We found that, whereas ABA-activated transcription factors *bZIP TF* (*BGIOSGA000430, BGIOSGA005551, BGIOSGA028831*) were upregulated in APO under drought conditions, *WRKY TF 67* and *WRKY TF 53* were downregulated both in APO and ENAPA. However, ABA regulated *Serine/threonine protein kinase*, *Dehydration-inducible SNF1-related protein kinase 2* (*BGIOSGA006276*), *Serine/threonine protein kinase*, and *ABA-activated protein kinase* (*BGIOSGA010254*) were only expressed and upregulated in APO grown under drought conditions. Within the receptor gene family, we found that *ABA receptor 9* (*BGIOSGA033490*) was uniquely downregulated in APO, whereas *ABA receptor 6* (*BGIOSGA010919*) was downregulated in all the three genotypes grown under drought. *ABA stress induced HVA22-like proteins* were also uniquely differentially expressed in APO. Interestingly, four *dehydration-responsive element binding protein 1 (DREB1)* also known as *C-repeat-binding factor (CBF)* encoding genes were downregulated only in APO and ENAPA. In summary, we observed the widest changes in ABA signalling gene expression occurred in APO.

### 3.6 Co-expression network analysis and key regulatory hubs identification

**Figure 7:**
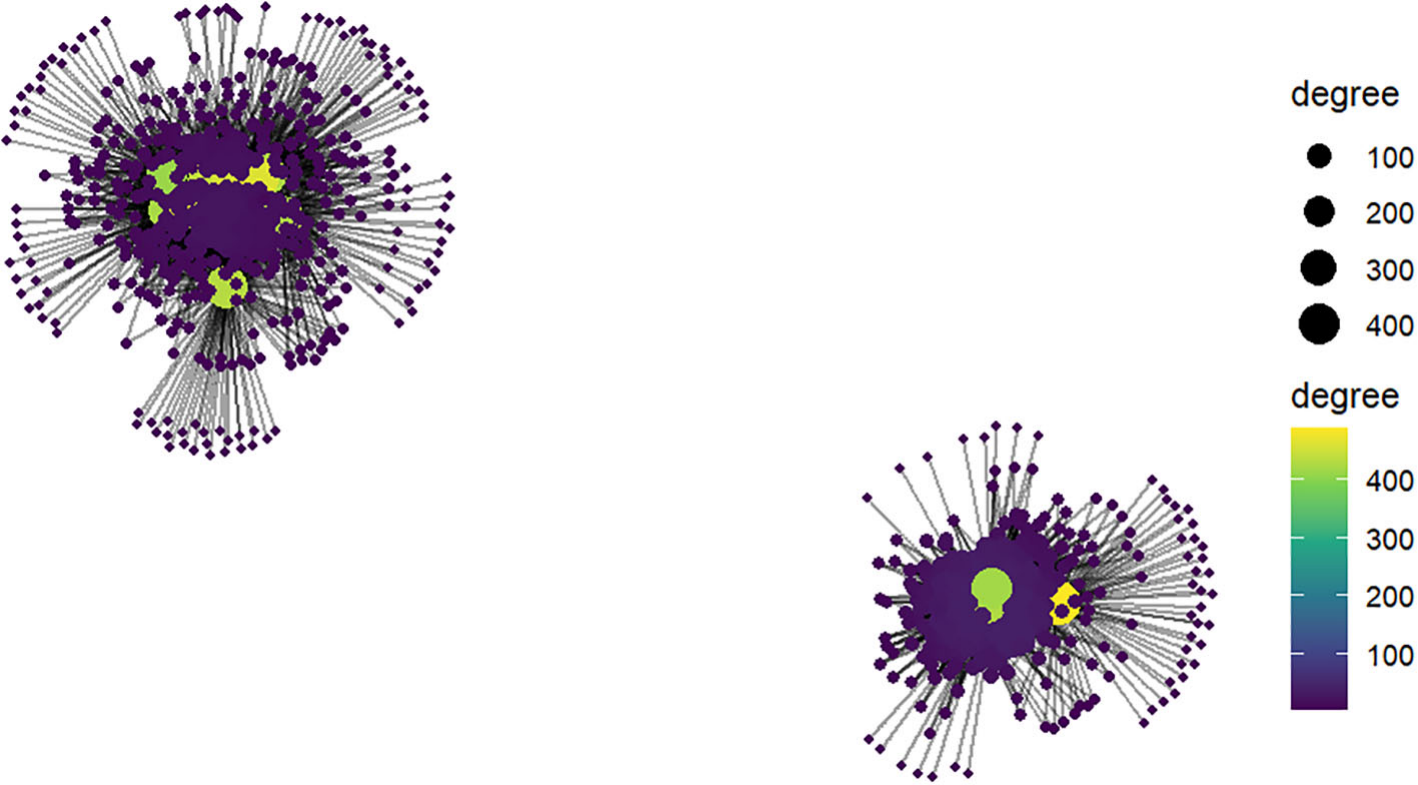
Regulatory hub gene network associated with drought response in rice. The network was inferred using mutual information-based approaches implemented in minet, and only the top 5% strongest regulatory interactions (835 hub genes) were retained to highlight the most robust connections. Network visualization highlighting the top regulatory hub genes identified from the co-expression and regulatory network analysis. Nodes represent genes and edges represent predicted regulatory interactions inferred from the expression data. Node size reflects gene connectivity (degree), indicating the number of interactions associated with each gene, while node color represents connectivity strength, with darker colors indicating lower connectivity and brighter colors indicating highly connected hub genes. The network is organized using a force-directed layout that groups genes according to their interaction density. Highly connected nodes located at the center of the network represent key regulatory hub genes, which potentially coordinate the expression of multiple downstream genes involved in drought response pathways. Two main network clusters are visible, reflecting the dominant regulatory structures identified within the trait-associated modules. The network was generated using the R packages igraph and ggraph.

From the total filtered genes, the top 20% most variable genes (n = 5044) were retained after normalization and used for subsequent network analysis. Selecting the most variable genes allowed to focus on those showing the greatest expression differences across samples, which are more likely to contribute to biologically meaningful regulatory patterns. The module-trait relationship analysis revealed that several modules were significantly associated with the studied drought stress and control conditions (p < 0.05). In particular, the module eigengenes ME2, ME1, ME8, and ME7, belonging mainly to the blue and turquoise modules, showed significant correlations with the traits (**Supplementary Additional File 5**). Within these trait-associated modules, a total of 3416 candidate hub genes were identified (**Supplementary Additional File 5**). To simplify the network and highlight the most relevant regulatory interactions, only the top 5% strongest edges were retained for visualization. This filtering resulted in a refined regulatory network containing 835 hub genes (**Figure 7**; **Supplementary Additional File 6**), allowing clearer identification of those most influential. Among these regulatory hubs, 30 genes stood out because of their exceptionally high connectivity, each interacting with more than 400 edges. Interestingly, the vast majority of these highly connected genes (28 genes) were located in the turquoise module, while only two genes were associated with the blue module, indicating turquoise module is a major regulatory centre within the network (**Figure 7**). Within this group, *BGIOSGA023969 (S-acyltransferase)* and *BGIOSGA038191 [NAD(P)-binding Rossmann-fold superfamily protein]*, both belonging to the turquoise module, showed the highest connectivity, with 488 and 462 edges, respectively. In addition to their high connectivity, these genes also exhibited strong associations with the shoot and root fresh weight under drought stress and control conditions, with gene significance (GS) values of 0.51 and 0.53, indicating that their expression patterns are closely linked to the studied drought stress and control conditions (**Supplementary Additional Files 5, 6**). Overall, the top 30 regulatory hub genes satisfied commonly accepted criteria for true hub genes, combining high gene significance (GS > 0.2) with strong module membership (kME > 0.8), (**Supplementary Additional File 5**). Functional enrichment analysis further revealed that these hub genes are significantly involved in biological processes related to hormone signalling, abiotic stress response, transcriptional regulation, and carbohydrate metabolism.

### 3.7 Quantitative real-time reverse transcription-PCR analysis

**Figure 8:**
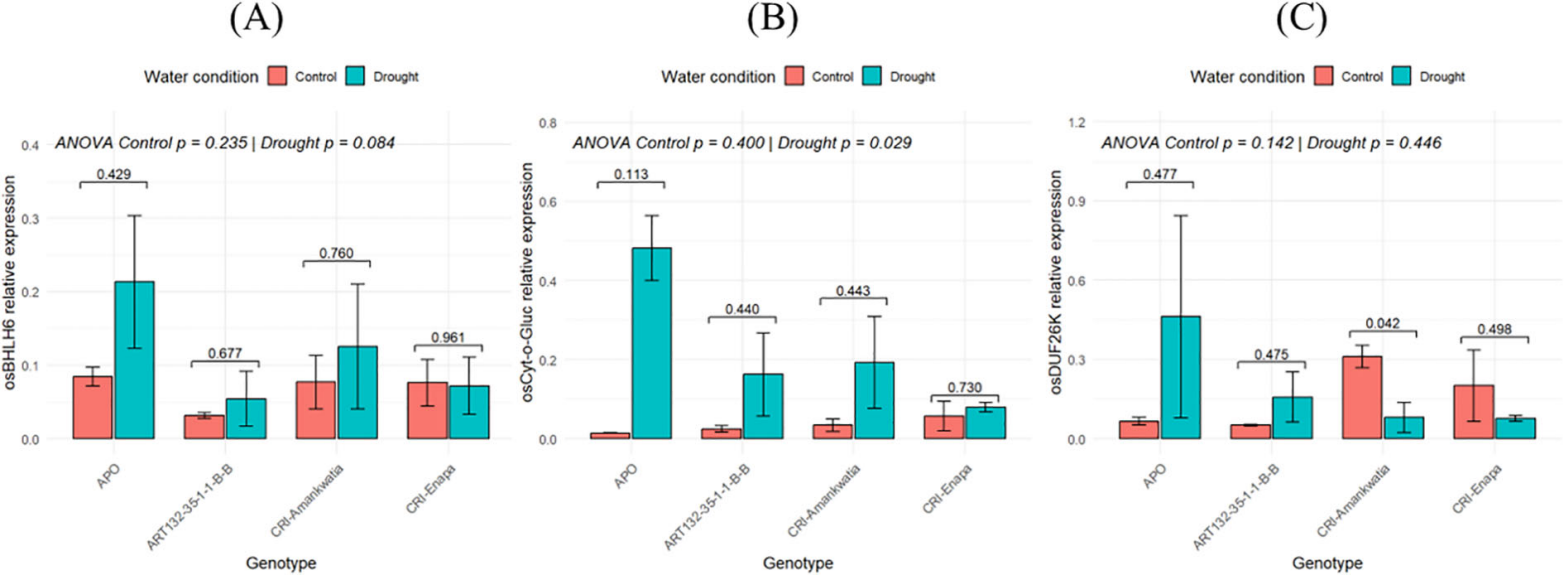
Expression of drought-responsive regulators in young plants of three rice varieties grown under drought and control conditions. Relative expression levels of **(A)***BHLH6*, **(B)***Cyt-o-Gluc*, **(C)***DUF26K*. Relative expression levels were determined using *RT-qPCR* with rice *Actin1* as a housekeeping gene. Data are means (± SD) of two biological replicates (n=6) each assessed in technical triplicates. Paired t-tests numeric p-values using brackets were used to assess the significance differences between control vs drought for each genotype. Statistically significant differences among the genotypes under control or drought are indicated with their corresponding p-value at the top of each figure as determined by two-way ANOVA followed by Duncan test. Statistically significant at 5% level of significance.

In parallel with our RNA-Seq analysis, we developed a targeted approach to determine how previously described rice drought-associated transcripts would accumulate in APO, Enapa, CRI-Amankwatia and ART32. After a literature search, we identified *Basic-helix-loop helix family protein*, *BHLH6*_ (LOC Os04g23550), *Cytokinin-O-glucosyltransferase 1*, *Cyt-o-Gluc* (LOC_Os05g08480), and *DUF26 kinases*, *DUF26K* (LOC_Os07g43560) as relevant genes for drought responses. We observed that under control conditions, *BHLH6* expression levels were similar in three varieties (APO, Enapa and Amankwatia), whereas ART32 accumulated approximately 3x less (**Figure 8A**). However, under drought conditions, we observed distinct responses, since *BHLH6* transcript levels showed a significant increase in APO (2.5x), whereas in Enapa, ART32, and CRI-Amankwatia these changes were minimal (0.9x, 1.7x, and 1.6x increase, respectively) (**Figure 8A**).

On the other hand, *Cyt-o-Gluc* expression levels were already different when the four varieties were grown under control conditions, with Enapa accumulating higher levels of this transcript and APO the lowest. However, under drought, *Cyt-o-Gluc* was significantly induced in APO (32.5x increase), while in ART32 and CRI-Amankwatia it was upregulated 6.5x and 5.5x under drought compared to control conditions, respectively. Similarly to *BHLH6*, *Cyt-o-Gluc* transcript levels were mostly unchanged in Enapa (1.4x increase in drought compared to control conditions) (**Figure 8B**).

Interestingly, *DUF26K* expression levels greatly differed between the four varieties. APO and ART32 accumulated low levels of *DUF26K* under control conditions oppositely to Enapa and Amankwatia that significantly showed a 2x and 3x increase, respectively, in its expression levels. However, when we compared drought and control conditions, we found that *DUF26K* was significantly upregulated in APO and ART32 (7.0x and 3.0x, respectively), but it was downregulated in Enapa (0.4x) and CRI-Amankwatia (0.3x) (**Figure 8C**).

Conclusively, we observed that there is significant difference in relative expression of drought responsive genes in all the varieties tested. However, similarly to what we found in our RNA-Seq analysis, APO showed the highest expression fold-change for the three drought-responsive genes (*BHLH6*, *Cyt-o-Gluc*, and *DUF26K*). Moreover, the relative constant expression of *BHLH6* and *Cyt-o-Gluc* in Enapa plants grown under drought and control conditions matched perfectly our RNA-Seq findings since these genes were not identified as DEGs.

In summary, our analysis identified specific regulators associated with exposure to drought during early developmental stages of the tested rice varieties previously screened for their tolerance under field conditions. We propose that these transcripts could be used as molecular markers to identify/validate drought resilient rice varieties both at early and later developmental stages.

## 4 Discussion

Breeding for drought tolerance is a challenging task due to the complexity of this trait which is just surpassed by grain yield. However, drought-tolerant varieties are key to the sustainable future of rice cultivation, especially in areas where water scarcity is increasing. Therefore, transcriptomic profiling of promising germplasm could contribute to identify drought-tolerant genotypes. Standard drought tolerance field trials focus mostly on later developmental stages, since grain filling and yield are some of the critical traits for farmers. However, knowledge collected from previous on-farm trials and participatory research projects with farmers showed that the occurrence of dry spells at early vegetative stages could lead up to 100% of seedling loss with the corresponding economic impact. To address this knowledge gap, we developed a comprehensive approach where varieties previously identified either as drought-tolerant (APO, Enapa), or sensitive (ART32, CRI-Amankwatia) were evaluated both at the physiological and molecular level under control and drought conditions at early developmental stages.

Our findings showed that APO and CRI-Amankwatia developed the highest shoot biomass under control conditions but were the most impacted under drought. On the other hand, Enapa and ART32 maintained their biomass both under control and drought conditions, suggesting that at early developmental stages, these two varieties are less affected by drought conditions. These findings seem to validate the growth/stress trade-off (Li et al., 2024; Takatsuji, 2017), since the varieties that grow more (e.g. APO) under optimal conditions fail to maintain their growth rates under stress. However, slower growing varieties (e.g., ART32) might be able to sustain their growth pattens under sub-optimal conditions and therefore will be less impacted by drought. When we performed a similar assessment of root biomass, we observed a slightly different response. In this case, APO, Enapa and CRI-Amankwatia are the best performers under control conditions; but under drought all the three varieties perform poorly, with APO being the most affected with the highest loss of root biomass. Interestingly, although ART32 displayed the lowest root biomass under control conditions, it also showed the lowest inhibition in root biomass under drought.

Together our data show that, at early vegetative stages (about 40 days) the rice varieties tested under drought conditions behaved somewhat differently from when they were assessed in the field at reproductive stage (Adjah et al., 2025). However, if we compare their performance under control conditions, APO is still the best variety. But when we compared their performance based on the relative shoot and root fresh weights, ART32 seems to behave better. This would be in agreement with previous studies showing a diminishing effect of drought stress on shoot and root weight across different developmental stages of the rice crop (Kou et al., 2022) and that the different rice genotypes could modulate their root-to-shoot ratio as a mechanism of drought adaptation (Kou et al., 2022; Xu et al., 2015).

It is also possible, that in our experimental set-up (50% soil water content), the severity of the drought stress might not be enough to induce significant differences among the genotypes for traits such as root fresh weight. However, these conditions seem to be sufficient to induce several gene expression changes in the varieties tested. Similar observations were previously made, since 40% of soil water content induced strong antioxidative response in APO (Melandri et al., 2021). The varieties tested also showed different degrees of transcriptional reprogramming under drought. APO underwent broader changes with 96.63% and 97.32% of uniquely regulated DEGs when compared to Enapa and ART32, respectively. On the other hand, only 20.68% of uniquely regulated DEGs were altered in Enapa compared to ART32. The large number of DEGs observed in the genotype APO under early vegetative-stage drought, despite its weaker phenotypic performance at this stage, suggests that this genotype may adopt a distinct strategy to cope with water deficit. APO has previously been reported as drought tolerant under reproductive-stage field conditions (Adjah et al., 2025). However, under our controlled laboratory setup, the drought conditions imposed were more homogeneous than in the field and, although they did not translate into major phenotypic differences, they promoted substantial transcriptional reprogramming. Therefore, the larger set of DEGs detected in APO could reflect some higher degree of transcriptional plasticity, where the plant activates a broad range of molecular pathways to buffer the effects of drought stress. Such genome-wide effects have been reported in several drought studies, where tolerant genotypes often display extensive activation of stress-responsive genes (Ahn et al., 2017; Zu et al., 2021; Chen et al., 2025). Moreover, this differential transcriptional behavior could indicate higher stress sensitivity in APO seedlings during the early vegetative stage to ensure the activation of protective pathways that will maintain their growth under water deficit. This pattern may point to a potential growth-defense trade-off, in which APO prioritizes stress-responsive regulatory processes over growth-related functions when exposed to drought during early development (De Tombeur et al., 2023; Cheaib et al., 2024). Although this interpretation is consistent with established models describing trade-offs between growth and stress defense in plants, further confirmation would require additional evidence, such as measurements of hormone dynamics, metabolic profiling, or biomass allocation patterns. Nevertheless, the strong transcriptional changes observed in APO seedlings under drought stress associated with their lower phenotype performance, suggests a distinct response strategy at this early development stage from that occurring later at the reproductive stage. Therefore, our findings emphasize the importance of the developmental context when interpreting genotype-specific responses to drought stress in rice.

This differential response also occurred when the GO categories associated with the drought-stress related DEGs were analysed in APO, Enapa and ART32. We identified phytohormone signalling (CKs, JA etc.), stress-responsive genes (e.g., *Dehydrins*, *LEA*), transcription factors (e.g., *bZIP*, *WRKY*), carbohydrate metabolism (e.g., S*WEET*), plant proteases and protease inhibitors, as well as transcripts involved in signalling transduction, as the most relevant categories. Interestingly, most of these groups were primarily represented in APO when compared with Enapa and ART32, confirming a previous report (Tiwari et al., 2021). However, Enapa also showed the significant modulation of stress-responsive DEGs such as *Dehydrin*s, *LEA* and *DREB*s which could associate with its higher stress tolerance over ART32, in agreement with a previous report (Tiwari et al., 2021).

Within the GO category “phytohormones”, we observed that several Auxin-responsive genes (e.g., *IAA5, Auxin-response factor 17*), as well as many ABA-induced transcripts (*HVA22-like proteins*, *ABA receptor 6* and *ABA receptor 9*) were downregulated in APO, while no significant changes were observed in Enapa and ART32. Similar Auxin/ABA gene expression modulation was reported in Arabidopsis, where the auxin repressors (*IAA5*, *IAA6*, *IAA19*) were positive regulators of drought-stress by promoting stomatal closure via the repression of the ABA-responsive transcription factor *WRKY63* (Salehin et al., 2019). Furthermore, the downregulation of *Auxin response factor* gene (*ARF*s) expression improves tolerance to stress (e.g., drought, salt) through increased stomatal closure and reduced leaf transpiration both in tomato (El Mamoun et al., 2023) and Arabidopsis (Meng et al., 2015; Salehin et al., 2019). It was also demonstrated that the rapid downregulation of auxin responses in dehydrating Arabidopsis seedlings resulted in retarded growth (Shani et al., 2017; Salehin et al., 2019). Possibly these responses could also explain the reduced shoot and root fresh weight observed in young APO plants grown under drought conditions, when compared to Enapa and ART32. Nevertheless, and despite this growth inhibition, APO plants managed to keep their leaves’ green which may be attributed to the up-regulation of *STAY-GREEN protein* expression (Kamal et al., 2019; Kumar et al., 2022).

*Gibberellin-induced A20/AN1 zinc-finger protein* (*BGIOSGA028955*) identified in this study is a negative regulator of GA (gibberellin) signalling and cell elongation; its downregulation could favour the expression of other plant hormones, especially ABA. Moreover, Gibberellin-induced A20/AN1 zinc-finger protein ZFP185 regulates plant growth and stress responses by inhibiting GA and ABA biosynthesis in rice (Zhang et al., 2016).

Lower Cytokinin (CK) content and signalling seem to play an antagonist role to ABA in drought tolerance (Son and Park, 2023). Possibly by increasing sensitivity to ABA and leading to a reduction in shoot growth (Li et al., 2020; Rhaman et al., 2025), which is a coping mechanism to drought (Cortleven et al., 2019). Therefore, the repression of most cytokinin (CK) related genes seen in APO could be associated with the upregulation of ABA signalling, providing a mechanism to improve drought tolerance in the field, and the shoot fresh weight reduction seen under laboratory conditions. Cytokinins are important to sustain stem cell division and shoot growth (Svolacchia and Sabatini, 2023) and these responses can be inactivated by cytokinin dehydrogenase (CKX) (Chen et al., 2020). Interestingly, we observed an upregulation of the *Cytokinin dehydrogenase 8* in APO under drought conditions, which could also explain the shoot growth inhibition observed.

Most of the jasmonate (JA) biosynthesis-related genes were repressed under drought conditions in APO, suggesting that their increase might be detrimental to rice growth as previously described (Harb et al., 2010; L. Huang et al., 2014; Park and Jeong, 2023). Moreover, the *Acyl-coenzyme A oxidase* gene that encodes a JA mediated enzyme was upregulated in APO, suggesting that antioxidant homeostasis could be important under drought (Shan and Liang, 2010).

Within the category “stress-responsive genes” we found an upregulation of the redox regulation related genes in APO when compared to Enapa and ART32. Redox regulation has been shown to promote drought-stress tolerance (Huang et al., 2014; Kaur et al., 2023) and this molecular mechanism could be at play in APO. A relevant osmoprotectant mechanism for drought in rice is proline accumulation which acts as an osmolyte, a metal chelator, an antioxidant, and a signalling molecule. Proline accumulation under stress conditions prevents electrolyte leakage and controls the accumulation of reactive oxygen species (ROS). This will prevent an oxidative burst, maintaining osmotic balance or cell turgor and membrane stabilisation (Hayat et al., 2012; Fu et al., 2018; Nasrin et al., 2020; Saddique et al., 2020; Frimpong et al., 2021). Proline aminopeptidase removes proline from proline-rich proteins and other related compounds, allowing its accumulation in the cells (Tarchevsky and Egorova, 2022). The upregulation of *proline aminopeptidase* transcript levels in APO could also be associated to its drought tolerance at later developmental stages.

Drought also induces *dehydrin* gene expression accordingly to plant growth stages (Zhang et al., 2013; Lv et al., 2017; Sena et al., 2018). Several *dehydrin* genes (*Dehydrin, Dehydrin Rab16C* and *Dehydrin Rab16D)* were upregulated in APO and Enapa, but not in ART32 which could indicate the protective role these dehydrin genes play in drought tolerant varieties which aligns with previous reports (Tiwari et al., 2021). Furthermore, it has been demonstrated that ABA-dependent and ABA-independent signalling pathways regulate the expression of dehydrin genes (Sun et al., 2021). Interestingly, we observed an upregulation of several ABA-signalling *bZIP* transcription factors in APO. *Group 3 LEA proteins* were also upregulated in all the varieties confirming their role in the dehydration response as previously suggested (Boswell et al., 2014). The induction of the *STAY-GREEN protein* gene delays leaf senescence and contributes to maintain green leaves, thereby improving biomass and yield under drought conditions (Kamal et al., 2019; Kumar et al., 2022). This transcript was highly induced in APO compared to Enapa and ART32, which could account for the greenness phenotype of plants from this variety (Kamal et al., 2019).

Within the GO category “transcription factors” (TFs) we found that *bZIP, GATA TF, ERF061, WRKY 47 MADS-box* were upregulated in APO which agrees with previous reports (Huang et al., 2014; Baldoni et al., 2021; Tiwari et al., 2021; Kaur et al., 2023; Park and Jeong, 2023). Possibly, the dynamic modulation of *bZIP* and WRKY TFs in APO and Enapa might also explain the difference in their tolerance to drought. Moreover, the significant upregulation under drought conditions of *Heat shock TF* genes (*BGIOSGA026537*) in APO and Enapa compared to ART32 could also account for the over-expression of drought-responsive genes in both varieties as high temperature is generally accompanied by drought.

In addition, we observed a significant enrichment in DEGs associated with the “plant proteases and proteases inhibitors” GO category, especially in APO. Previous reports suggest that plant proteases, and some protease inhibitors, could help maintain cellular homeostasis under drought conditions by regulating stomatal closure, promoting the induction of ABA-related stress genes, and the maintenance of relative water content (D’Ippólito et al., 2021; Moloi and Ngara, 2023). Moreover, drought-tolerant genotypes seem to show an induction of protease inhibitors, as they tend to inhibit protein degradation, while drought-sensitive genotypes would accumulate proteases (Moloi and Ngara, 2023). However, this could not be ascertained in this study, since only one *cysteine proteinase inhibitor* (*BGIOSGA017804*) was differently expressed and upregulated in APO (drought-tolerant genotype) and another *cysteine proteinase inhibitor* (*BGIOSGA030147*) in ART32 (drought-sensitive genotype).

We also identified several DEGs within the “carbohydrate metabolism” GO category. Among those we found *Glycosyltransferase*, *UDP-glucosyl transferase (UGT) family proteins*, and *malate dehydrogenase* which were differentially expressed under drought only in APO, suggesting an osmotic adjustment in this variety in agreement with previous reports (Liu et al., 2010; Huang et al., 2014). Moreover, the dynamic expression of the *Bidirectional sugar transporter SWEET Gene Family* with the upregulation of *SWEET6b* and *SWEET6a*, and the downregulation of *SWEET11* and *SWEET14* in APO, compared to Enapa and ART32, suggests that APO plants have different ways to cope with drought (Gautam et al., 2022; Ji et al., 2022). Interestingly, we found that the senescence marker gene *SWEET15* was upregulated in all the three varieties grown under drought (Gautam et al., 2022). This suggests that carbohydrate remobilization via phloem loading was probably in place, similar to the processes that occur during leaf senescence in different species (Seo et al., 2010; Chen et al., 2015).

Several protein kinases encoding transcripts (e.g., *Serine/threonine protein kinases, RKs*, *CDPK 29*, *ATP-dependent 6-phosphofructokinase 2, UMP-CMP kinase*, *Diacylglycerol kinase)* have been associated with drought-stress responses (Marshall et al., 2012; Huang et al., 2014; Chen et al., 2021; Kong et al., 2023; Movahedi et al., 2023), mostly due to their involvement in signal transduction. We found that *Phosphoglycerate kinase* was upregulated in all the three genotypes, confirming previous reports of its induction under combined drought and nitrogen deficiency stress (Li and Wang, 2023). The upregulation of RKs in APO, aligns with the suggested mode of action of this gene family, since they perceive and then transmit extracellular signals (e.g., drought) via phosphorylation cascades which will initiate specific responses to those abiotic stresses (Chen et al., 2021).

In addition to GO and gene level analysis, a system level analysis using a co-expression network approach was used to identify key regulatory modules and hub genes associated with drought response in rice. By focusing on the most variable genes across samples, our module-trait relationship analysis highlighted the turquoise and blue modules as significantly associated with the studied conditions. This finding suggests a connection between these modules and those genes responding strongly to the abiotic stress. Moreover, the relevance of the turquoise module in terms of hub gene abundance and connectivity highlights its role as a central regulatory cluster controlling drought-responsive transcriptional programs. Similar patterns have been reported in previous network studies where a limited number of modules concentrate most regulatory interactions, reflecting coordinated transcriptional regulation of stress responses (Wilkins et al., 2016; Smita et al., 2019; Shanks et al., 2022; Zhang et al., 2023).

The identification of 3416 candidate hub genes within these modules further underscores the complexity of transcriptional regulation during drought stress. However, filtering the network to retain only the strongest interactions revealed a core set of 835 top connected regulatory genes. Such network refinement strategies are commonly used in transcriptome studies to highlight biologically meaningful interactions while minimizing noise inherent to large-scale gene expression datasets.

The identification of 30 highly connected regulatory hub genes, each interacting with more than 400 network edges with their predominance within the turquoise module, further confirms this module acts as a key regulatory center within the drought-response network. Highly connected hub genes are often considered potential master regulators because perturbation of their activity can influence the expression of many downstream genes. In agreement with this concept, genes with high connectivity frequently correspond to transcription factors or signaling components that modulate stress-responsive pathways (Shanks et al., 2022).

Among the identified hub genes, *BGIOSGA023969* and *BGIOSGA038191* showed the highest connectivity and strong correlations with the studied phenotype. In co-expression networks, such genes are particularly valuable candidates for further functional characterization, as they may represent key regulatory nodes controlling stress-responsive transcriptional cascades.

The functional enrichment analysis provides additional biological insights into the regulatory mechanisms captured by the network. Our identified hub genes were significantly enriched in pathways related to hormone signaling, abiotic stress responses, transcriptional regulation, and carbohydrate metabolism, all of which are well-established components of plant drought adaptation. Hormone-mediated signaling pathways, especially those involving abscisic acid (ABA), play central roles in modulating plant responses to water deficit. ABA signaling regulates a wide range of physiological processes, including stomatal closure, osmotic adjustment, and activation of stress-responsive genes. Previous studies have shown that ABA-dependent transcription factors (TFs) such as NAC, AP2/ERF, and bZIP family members regulate downstream genes involved in osmotic protection and oxidative stress mitigation in rice under drought conditions (Ma et al., 2024). Several TFs, including OsNAC29a and members of the AP2/ERF family such as OsERF74, have been identified as key regulators of drought tolerance in rice by modulating ABA signaling pathways and activating stress-responsive genes (Lu et al., 2025; Luo et al., 2026). These findings are consistent with the enrichment of transcriptional regulation and hormone signaling pathways observed in our network analysis such as *BGIOSGA005551* and B*GIOSGA000430 (bZIP transcription factors)*, and *BGIOSGA028819 (HVA22-like protein)*, suggesting that similar regulatory mechanisms may underline the expression patterns identified in this study.

In addition to transcriptional regulation, the enrichment of genes associated with carbohydrate metabolism highlights the importance of metabolic adjustment during drought stress. Under water deficit conditions, plants often alter carbohydrate allocation and osmolyte accumulation to maintain cellular homeostasis and protect cellular structures (Kim et al., 2017). These metabolic adjustments contribute to maintaining photosynthetic efficiency and energy balance during stress conditions. Moreover, TFs involved in drought responses frequently coordinate both regulatory and metabolic pathways to optimize plant survival under environmental stress.

In addition to our genome-wide transcriptional and co-expression analysis, we also assessed targeted transcriptional responses associated with drought experienced at early development stages of these elite varieties. Our findings show that *BHLH6*, *Cyt-o-Gluc* and *DUF26K*, previously described as drought responsive genes (Huang et al., 2014; Tiwari et al., 2021) were also differentially expressed in APO, Enapa and ART32 in our RT-qPCR analysis. These results agree with our RNA-seq findings, showing a clear correlation between the two methodologies, and highlighting the transcriptional re-programming occurring in APO and Enapa (although in a smaller scale) when grown under drought conditions (Kaur et al., 2023; Park and Jeong, 2023).

Taken together, the identification of highly connected hub genes and trait-associated co-expression modules provides valuable insights into the development of drought-resilient rice varieties. Due to hub genes’ central role within regulatory networks, they are likely to induce broad influence on downstream stress-responsive pathways. As such, the candidate genes highlighted in this study represent promising targets for further functional validation and for the development of molecular markers that could accelerate breeding for drought tolerance. Integrating these regulatory candidates into modern breeding pipelines through marker-assisted selection, genomic selection, or genome-editing approaches could help enhance the efficiency of introgressing drought resilient traits into elite cultivars. On the other hand, by revealing regulatory genes associated with hormone signaling, transcription regulation, and metabolic adjustment, this study contributes to a growing body of knowledge aimed at strengthening crop resilience under water-limited conditions. Future works combining transcriptomic network analyses with physiological studies and field-based validation will be essential to translate these discoveries into practical breeding outcomes.

## 5 Conclusion and implications for rice breeding and production in sub-Saharan Africa

In this study, we followed a parallel approach to characterise drought-induced phenotypical and transcriptional responses of four elite rice varieties at early developmental stages. These four contrasting genotypes were previously evalauted for their drought tolerance under field conditions, where APO and Enapa were described as drought-tolerant, whereas ART32 and CRI-Amankwatia were drought-sensitive. Our approach showed that drought responses vary at early development, and different drought-tolerant varieties engage into specific molecular responses. By characterising the transcriptome of these plants, we aimed to uncover the molecular mechanisms behind their tolerance/sensitivity. Therefore, we propose that breeding for drought tolerance in rice should not only focus on late vegetative and reproductive stages but also include an assessment of drought resilience at an early vegetative stage (between 10 to 30 days after sowing or transplanting). The occurrence of drought-stress (between two to four weeks of dry spells) often desiccates young rice seedlings resulting in 100% crop failure and enormous economic loss for rice growers, especially small holder farmers. Recurrent droughts in upland rice production across west Africa are increasing in frequency, and early vegetative seedling failure is one of the numerous challenges (including soil pauperisation, weed pressure, lack of supplemental irrigation) that growers currently face. Considering this, our findings provide evidence of available genetic diversity (e.g., DEGs) which could contribute to improve drought tolerance, both at early and late developmental stages. Particularly, our findings identified 835 highly connected hub genes, among which 30 genes exhibited exceptionally high connectivity, suggesting potential central roles in coordinating stress-responsive gene expression, and highlighting them as valuable candidates for further analysis. In addition, our results also emphasize the need to assess rice drought tolerance at early vegetative stages, and this information should be included in the passport data of the released varieties to help rice farmers make informed decisions.

## Data Availability

The row dataset generated during and/or analysed during the current study are available in the Mendeley data (DOI: 10.17632/3cg2gp86rn.1, https://data.mendeley.com/datasets/3cg2gp86rn/1).
