# Supplementary material for: Shoot transcriptome and co-expression network analysis of African rice cultivars identify drought-tolerance hub genes and growth stage-dependent trade-offs
Source: Front Plant Sci. 2026 Apr 15;17:1802527. doi: 10.3389/fpls.2026.1802527 (PMC13126551; doi:10.3389/fpls.2026.1802527)
Supplement: Supplementary file 1 [file SupplementaryFile1.zip › Supplementary files/Additional file 1.docx]

Additional file 1_S1. Mean performance of 4 rice genotypes evaluated for chlorophyll content Index (**A**), spikelet fertility score (**B**), days to flowering (**C**), grain yield per plant (**D**), leaf rolling score (**E**), recovery from drought (**F**) at early reproductive stage under drought-stress and non-stress at the CSIR-Crops Research Institute, Ghana, in 2021-2022. Data presented are means ±SE (n=15 accounting for 5 plants per replicate, 3 replicates). From left to right, genotypes are classified from best to worst performance, respectively, based on delay in days to flowering, leaf rolling score, recovery from drought, and the relative value of chlorophyll content Index, spikelet fertility score, grain yield per plant, plant height. Delay in days to flowering is calculated as number of days to flowering under drought-stress - number of days to flowering under non-stress conditions per genotype. Relative values were calculated as value under drought-stress/value under non-stress conditions per genotype. For the leaf rolling score and recovery from drought, the lower the score, the better the performance of the genotype under drought-stress. Leaf rolling was scored as follows: 0 (leaves healthy), 1 (leaves start to fold (shallow), 3 (leaves folding (deep V-shape), 5 (leaves fully cupped (U-shape), 7 (leaves’ margins touching (0-shape), and 9 (leaves tightly rolled (V-shape). The scoring of spikelet fertility was as follows: 1 (more than 80%), 3 (61–80%), 5 (41–60%), 7 (11–40%), and 9 (less than 11%). Plants recovery from drought scores were taken after 10 days following watering as 1 (90–100% of plants recovered), 3 (70–89%), 5 (40–69%), 7 (20–39%), and 9 (0–19%). Days to 50% flowering (DTF) were recorded when 50% of the plants in each plot showed flowering.


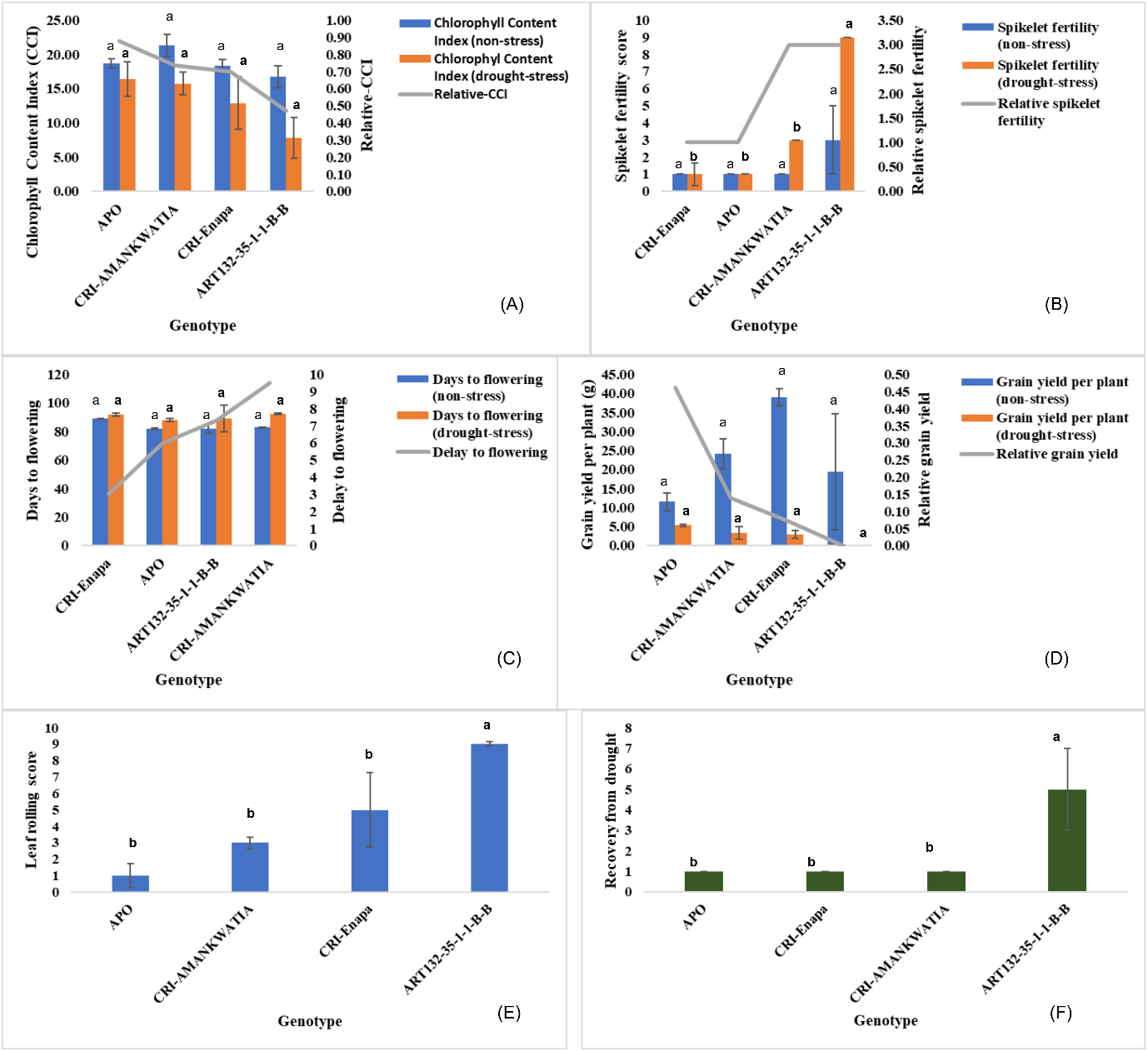


Additional file 1_S2. Primers used for the *RT-qPCR* amongst the rice genotypes evaluated under drought conditions

| S/N | Gene Name | Gene ID | Forward Primer (5′→ 3′) | Reverse Primer (5′→ 3′) |
| --- | --- | --- | --- | --- |
| 1 | *Transciption factor BHLH6* | LOC_Os04g23550 | TGCGAGGCAACGATTTAGT | GCTGGACCACCTTATTATTCAT |
| 2 | *Cytokinin-O-glucosyltransferase 1* | LOC_Os05g08480 | CATCAGTGGCATCAGAAGTCG | GCAGCATTATTATTGCCATTGT |
| 3 | DUF26 kinases | LOC_Os07g43560 | AACCATACCCTGTAACCGA | CCTGAAAATACAATAAATACACAA |
| 4 | *Actin1* | LOC_Os03g50885 | TTGCTGACAGGATGAGCAAG | TGGAATGTGCTGAGAGATGC |

Additional file 1_S3. RNA concentration observed amongst the rice genotypes evaluated under drought-stress and non-stress in pots at University College Cork (UCC), Cork, Ireland in 2023

| RNA quantification technique | | NANODROP | | QUBIT | |
| --- | --- | --- | --- | --- | --- |
| Rep | Genotype | RNA-NS (ng/µL) | RNA-DS (ng/µL) | RNA-NS (ng/µL) | RNA-DS (ng/µL) |
| 1 | APO | 834.80 | 1025.70 | 1176 | 1624 |
| 2 | APO | 883.30 | 1700.50 | 776 | 884 |
| 3 | APO | 997.9 | 699.40 | 1225 | 856 |
| 1 | ART32 | 1430.20 | 1523.00 | 1364 | 1312 |
| 2 | ART32 | 2141.20 | 960.00 | 1364 | 412 |
| 3 | ART32 | 1756.00 | 1487.20 | 896 | 1484 |
| 1 | CRI-Amankwatia | 1017.30 | 1222.50 | - | - |
| 2 | CRI-Amankwatia | 856.30 | 1432.00 | - | - |
| 3 | CRI-Amankwatia | 846.00 | 298.50 | - | - |
| 1 | Enapa | 1533.60 | 1011.70 | 700 | 1104 |
| 2 | Enapa | 1232.50 | 1329.50 | 1392 | 776 |
| 3 | Enapa | 1361.80 | 2125.80 | 1056 | 1532 |

RNA-NS: RNA concentration in ng/µL under non-stress; RNA-DS: RNA concentration in ng/µL under drought-stress

Additional file 1_S4. Assessment of RNA quality by denaturing gel electrophoresis from the rice genotypes evaluated under drought stress and non-stress in pots at University College Cork (UCC), Cork, Ireland in 2023. **(1)** APO/NS/R1; **(2)** APO/DS/R1;**(3)** APO/NS/R2; **(4)** APO/DS/R2; **(5)** APO/NS/R3; **(6)** APO/DS/R3; **(7)** Enapa/NS/R1; **(8)** Enapa/DS/R1;**(9)** Enapa/NS/R2; **(10)** Enapa/DS/R2; **(11)** Enapa/NS/R3; **(12)** Enapa/DS/R3; **(13)** ART32/NS/R1; **(14)** ART32/DS/R1;**(15)** ART32/NS/R2; **(16)** ART32/DS/R2; **(17)** ART32/NS/R3; **(18)** ART32/DS/R3. Non-stress=NS; Drought-stress=DS; R=Replicate.


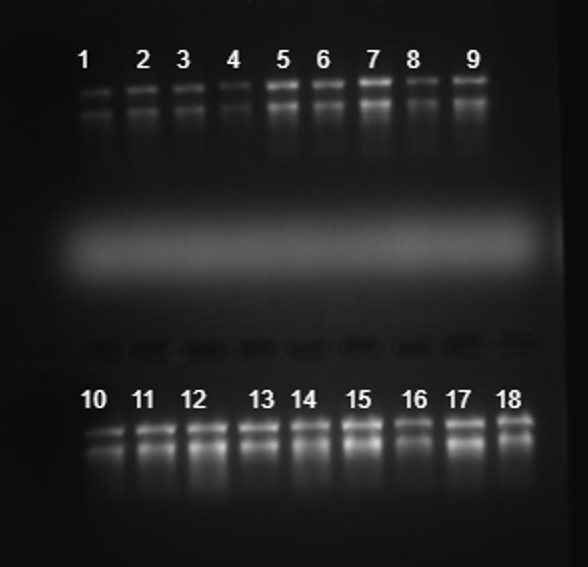


Additional file 1_S5. The total number of input reads, the percentage of reads uniquely mapped to the reference genome, the percentage of reads mapping to multiple locations, and the percentage of unmapped reads among three rice genotypes evaluated under drought-stress and non-stress in pots at University College Cork (UCC), Cork, Ireland in 2023.

| **Sample**  **Name** | **Total input reads** | **Uniquely mapped**  **reads** | **Reads mapped to**  **multiple/too many loci** | **Unmapped**  **reads** |
| --- | --- | --- | --- | --- |
| APO-CT-R1 | 41029315 | 37928505 (92.44%) | 1199096 (2.92%) | 1900309 (4.64%) |
| APO-D-R1 | 36420609 | 33712720 (92.56%) | 1017772 (2.79%) | 1688182 (4.64%) |
| APO-CT-R2 | 42488182 | 38820410 (91.37%) | 1324632 (3.12%) | 2339519 (5.5%) |
| APO-D-R2 | 44013994 | 40451238 (91.91%) | 1294316 (2.94%) | 2265281 (5.15%) |
| APO-CT-R3 | 34082054 | 30967755 (90.86%) | 1251828 (3.68%) | 1859894 (5.46%) |
| APO-D-R3 | 40152398 | 36609411 (91.18%) | 1116457 (2.78%) | 2423075 (6.04%) |
| Enapa-CT-R1 | 36121440 | 32986172 (91.32%) | 1121688 (3.1%) | 2010034 (5.57%) |
| Enapa-D-R1 | 33351394 | 30647005 (91.89%) | 1056469 (3.17%) | 1645559 (4.94%) |
| Enapa-CT-R2 | 30131368 | 27580098 (91.53%) | 917825 (3.05%) | 1630496 (5.41%) |
| Enapa-D-R2 | 33477613 | 30402045 (90.81%) | 1036023 (3.09%) | 2035601 (6.08%) |
| Enapa-CT-R3 | 41444684 | 37780172 (91.16%) | 1326684 (3.2%) | 2333754 (5.63%) |
| Enapa-D-R3 | 34745117 | 31712714 (91.27%) | 1110909 (3.2%) | 1918292 (5.52%) |
| ART32-CT-R1 | 30874245 | 27764333 (89.93%) | 1283329 (4.15%) | 1823205 (5.91%) |
| ART32-D-R1 | 35602395 | 32626644 (91.64%) | 1052119 (2.95%) | 1920295 (5.4%) |
| ART32-CT-R2 | 36417281 | 33167696 (91.08%) | 1171993 (3.22%) | 2073512 (5.69%) |
| ART32-D-R2 | 42376594 | 38503878 (90.86%) | 1373660 (3.24%) | 2495066 (5.89%) |
| ART32-CT-R3 | 39661001 | 36205277 (91.29%) | 1246360 (3.14%) | 2205953 (5.56%) |
| ART32-D-R3 | 34596037 | 31644391 (91.47%) | 1087242 (3.15%) | 1860551 (5.37%) |
